# Interleukin-1α -899 (+4845) C→T polymorphism is not associated with aggressive periodontitis susceptibility: A meta-analysis based on 19 case-control studies

**DOI:** 10.3892/br.2014.240

**Published:** 2014-02-19

**Authors:** WAN-FEN WANG, JUN SHI, SHAO-JUAN CHEN, YU-MING NIU, XIAN-TAO ZENG

**Affiliations:** 1 Department of Stomatology, Taihe Hospital, Hubei University of Medicine, Shiyan, Hubei, P.R. China; 2 Department of Stomatology, Shiyan Maternal and Child Health Hospital, Hubei University of Medicine, Shiyan, Hubei, P.R. China; 3 Center for Evidence-based Medicine and Clinical Research, Taihe Hospital, Hubei University of Medicine, Shiyan, Hubei, P.R. China

**Keywords:** aggressive periodontitis, susceptibility, interleukin-1α, polymorphism, meta-analysis

## Abstract

A number of published studies investigated the association between interleukin-1α (IL-1α) −899 (+4845) C→T polymorphism and susceptibility to aggressive periodontitis (AgP). However, the results from different studies are controversial. This study was conducted to further investigate the association between IL-1α −899 (+4845) C→T polymorphism and AgP using a meta-analysis. A search was conducted through PubMed up to May 1, 2013 and a total of 19 relevant case-control studies were identified. The results of this meta-analysis demonstrated that IL-1α −899 (+4845) C→T polymorphism is not associated with susceptibility to AgP under allele T vs. C [odds ratio (OR)=1.00, 95% confidence interval (CI): 0.88–1.14, P=0.98; I^2^=28.86%] or allele A vs. C comparison (OR=0.99, 95% CI: 0.85–1.14, P=0.85; I^2^=33.66%). The subgroup analyses based on ethnicity, source of controls and Hardy-Weinberg equilibrium (HWE) also revealed no such association. There existed a weak publication bias (Egger’s test P=0.02). In conclusion, based on the currently available evidence, there is no association between IL-1α −899 (+4845) C→T polymorphism and susceptibility to AgP.

## Introduction

Periodontitis is an inflammatory disease of the supporting tissues of the teeth (1) and is considered to be a risk factor for several systemic conditions (2), such as cardiovascular disease (3,4), diabetes (5) and chronic obstructive pulmonary disease (6). Periodontitis is currently classified as two main forms: chronic periodontitis (CP) and aggressive periodontitis (AgP). AgP is less prevalent compared to CP, but is associated with more rapid attachment loss and bone destruction (7). The pathophysiology of this disease may be determined by a variety of microbial, environmental, genetic and behavioural factors and systemic diseases (8). However, for patients with a reasonably good oral hygiene who develop this condition (7), AgP is considered to be a genetically inherited disease (9). Several family-based studies demonstrated that AgP is an autosomal recessive disorder (10–13), indicating a missense mutation of the cathepsin C gene in the 11q14 chromosome as being involved in the development of this disease (14).

Various candidate genes have been investigated for association with AgP and polymorphisms in the interleukin-1α (IL-1α) gene were suggested to affect the transcription of IL-1α, with −899 (+4845) C→T identified as one of the relevant polymorphisms. Walker *et al* (15) first reported that the IL-1α −899 (+4845) C→T polymorphism was not associated with the risk of AgP in African-American patients. Since then, a number of studies were conducted to investigate the association between this polymorphism and the risk of AgP. However, those studies had two limitations: i) consistent results could not be obtained due to the selection criteria of patients and controls and their ethnic origin; and ii) the majority of those studies included a limited number of patients (≤40). Therefore, the aim of this study was to investigate the association between IL-1α −899 (+4845) C→T polymorphism and the risk of AgP by performing a meta-analysis of previously conducted studies.

## Materials and methods

### Eligibility criteria

The inclusion criteria were as follows: i) studies that investigated the association between the IL-1α −899 (+4845) C→T polymorphism and susceptibility to AgP; ii) the study design was case-control and the diagnostic criteria of AgP were clearly reported; iii) the AgP patients were free of any other systemic disease and the control subjects were healthy or periodontitis-free; iv) the studies reported the odds ratios (ORs) and associated 95% confidence intervals (CIs) or the number of individual genotypes in the case and control groups, or the data required for their calculation; and v) the publication language was English or Chinese.

This meta-analysis was conducted in concordance with the Meta-analysis Of Observational Studies in Epidemiology (MOOSE) guidelines (16).

### Search strategy

The search terms ‘polymorphism’ or ‘mutation’ or ‘variant’, ‘interleukin-1’ or ‘IL-1’ and ‘periodontitis’ or ‘periodontal disease’ were used to conduct a search through PubMed up to May 1, 2013. The reference lists of the included studies and recent reviews were manually searched to identify additional relevant studies.

### Data extraction

According to the prespecified selection criteria, two authors independently selected the studies and extracted the following data: surname of first author, year of publication, country of origin and ethnicity, source of controls, number and genotyping distribution of cases and controls, OR and its 95% CI, genotyping method and Hardy-Weinberg equilibrium (HWE) for controls. Any disagreements were resolved by consulting a third author.

### Statistical analysis

We calculated the ORs and corresponding 95% CIs for the rare and common alleles. Heterogeneity among included studies was detected using I^2^ statistics (17). The value of I^2^≤40% was considered to indicate no significant heterogeneity and the fixed effects model was used; otherwise, the random effects model was used. We also performed subgroup analyses based on ethnicity, source of controls and the HWE for controls. A sensitivity analysis was performed by excluding one study at a time. The publication bias was assessed by funnel plot analysis and the Egger’s linear regression test (18). All the analyses were performed using Comprehensive Meta-Analysis software, version 2 (Biostat, Inc., Englewood, NJ, USA) (19) and the P-values were two-sided.

## Results

### Study characteristics

**Figure 1:**
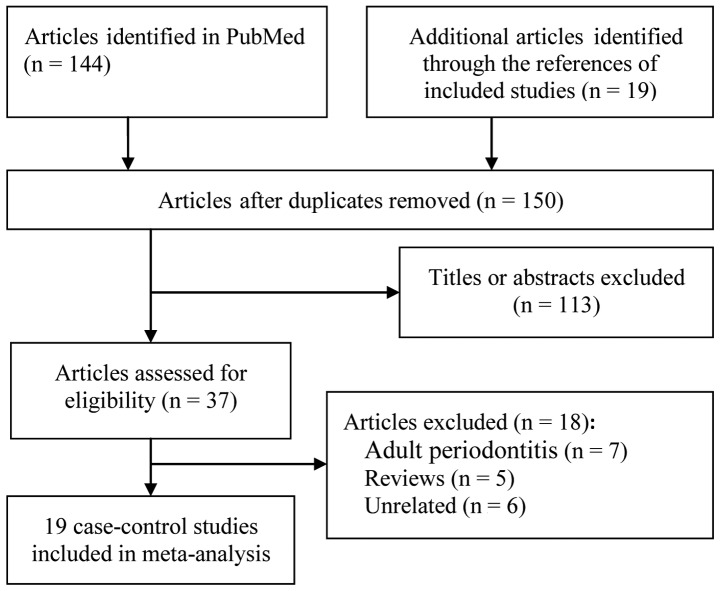
Flow chart of the study selection process, from identification to final inclusion.

The primary search resulted in the identification of 163 publications. Finally, 19 case-control studies, including a total of 1,266 AgP patients and 2,134 healthy controls, were deemed as eligible for inclusion in this meta-analysis (15,20–37). The study selection process is shown in Fig. 1.

Of the 19 studies, 14 investigated probands of Caucasian (20,22,24,26–33,35–37), 4 of Asian (21,23,25,34), and 1 of African-American origin (15). Only 1 study was not in HWE (27) and 1 study reported ORs and 95% CIs for the rare and common alleles (32). The main characteristics of the identified studies are summarized in Table I.

### Meta-analysis

**Figure 2:**
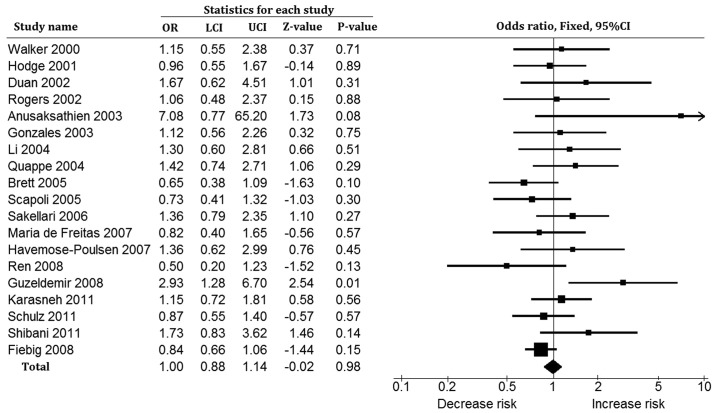
Forest plot for IL-1α −899 (+4845) C→T associated with risk of aggressive periodontitis in T vs. C comparison (fixed effects model). OR, odds ratio; CI, confidence interval; LCI, lower CI; UCI, upper CI.

**Figure 3:**
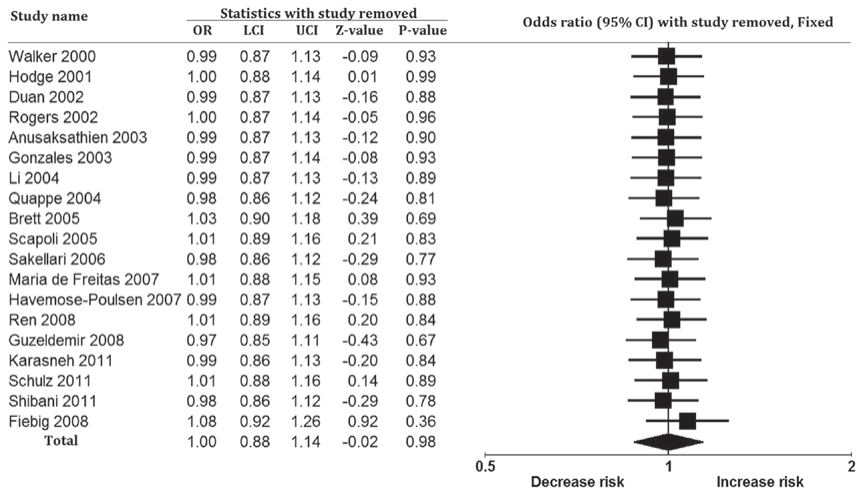
Sensitivity analysis by detecting any single study each time in T vs. C comparison (fixed effects model). OR, odds ratio; CI, confidence interval; LCI, lower CI; UCI, upper CI.

Overall, the rare and common alleles provided no evidence supporting an association between the IL-1α −899 (+4845) C→T polymorphism and susceptibility to AgP (OR=1.00, 95% CI: 0.88–1.14, P=0.98 for the T vs. C allele and OR=0.99, 95% CI: 0.85–1.14, P=0.85 for the C vs. T allele; Fig. 2). The subgroup analyses for ethnicity, source of controls and HWE, also revealed no such association. The sensitivity analysis, performed by excluding studies one at a time, reached a similar conclusion (Fig. 3). The results of the meta-analysis are presented in Table II.

### Publication bias

**Figure 4:**
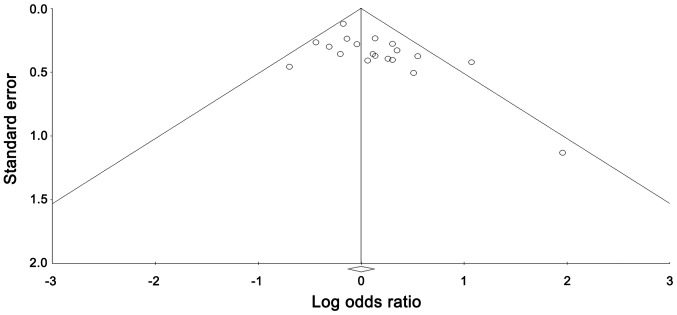
Funnel plot for the assessment of publication bias in T vs. C comparison (fixed effects model).

A weak publication bias was observed in this meta-analysis (Fig. 4), as evidenced by Egger’s test (P=0.02), also shown in detail in Table II.

## Discussion

The early onset form of periodontitis (juvenile, post-juvenile, post-adolescent) is less prevalent, but is associated with more rapid bone destruction at a relatively young age and is therefore termed ‘AgP’ (32). Unlike CP, AgP is likely a genetically inherited disease (9). Numerous studies previously evaluated the association between IL-1α −899 (+4845) C→T polymorphism and AgP, although the reported results are inconsistent (Fig. 2). In addition, the credibility of the results from a single case-control study is questionable due to the relatively limited sample size. Meta-analysis has being widely used in genetic association studies due to its advantage of overcoming this limitation (38–40). The present meta-analysis, based on 19 case-control studies, aimed to provide a comprehensive analysis of the association between IL-1α −899 (+4845) C→T polymorphism and susceptibility to AgP. Our results indicated that there is no such association, either under T vs. C or C vs. T allele comparison. These results are consistent with those of the largest-sample and multicenter study, including a total of 415 AgP patients and 874 healthy controls (32), but not with the results of the smallest-sample and single-center study, which included 31 AgP patients and 31 healthy controls (33). This finding may provide evidence that a meta-analysis has the advantage of overcoming the limitations of a single case-control study by enlarging the sample size.

To further investigate the association between IL-1α −899 (+4845) C→T polymorphism and susceptibility to AgP, subgroup analyses by ethnicity, source of controls and HWE were performed. All the results were consistent with those of the overall analysis, indicating no role for ethnic background or environment. Therefore, AgP is likely a genetically inherited disease (9) and it was concluded from our results that the C or T allele did not affect the risk of AgP.

There were some limitations to our meta-analysis. First, the patient samples of the majority of the included studies were limited (≤40). Although this may be attributed to the low prevalence of AgP, a small sample size is associated with the possibility of bias. Second, despite thorough attempts to collect relevant studies, publication bias also existed. This may result from the language limitation to English and Chinese, or from negative results not being published. Third, due to the lack of data on origin in the included studies, we were unable to assess the gene-environment interactions, which may affect the susceptibility to AgP. Finally, the negative association between IL-1α −899 (+4845) C→T polymorphism and susceptibility to AgP should be interpreted with caution.

In conclusion, our meta-analysis, based on a total of 1,266 AgP patients and 2,134 healthy controls, demonstrated that the IL-1α −899 (+4845) C→T polymorphism is not associated with the risk of AgP. However, due to the abovementioned limitations of the present study, our results may be considered as inconclusive.

## Figures and Tables

**Table I:** Characteristics of the studies included in the meta-analysis.

First author (year)	Country (ethnicity)	Cases			Source of controls	Controls			HWE	Refs.
	
		Total	G	T		Total	G	T		
Walker (2000)	USA (Negroid)	37	62	12	Population	104	178	30	0.89	(15)
Hodge (2001)	UK (Caucasian)	56	74	38	Hospital	56	73	39	0.902	(20)
Duan (2002)	China (Asian)	20	34	6	Hospital	94	170	18	0.305	(21)
Rogers (2002)	Australia (Caucasian)	21	31	11	Population	60	90	30	0.61	(22)
Anusaksathien (2003)	Thailand (Asian)	26	48	4	Hospital	43	85	1	0.94	(23)
Gonzales (2003)	Germany (Caucasian)	43	66	20	Population	47	74	20	0.103	(24)
Li (2004)	China (Asian)	122	226	18	Mixed	95	179	11	0.55	(25)
Quappe (2004)	Chile (Caucasian)	36	52	20	Hospital	75	118	32	0.33	(26)
Brett (2005)	UK (Caucasian)	50	73	27	Population	103	131	75	0.02	(27)
Scapoli (2005)	Italy (Caucasian)	40	60	20	Population	96	126	60	0.88	(28)
Sakellari (2006)	Greece (Caucasian)	40	51	29	Mixed	100	141	59	0.53	(29)
Maria de Freitas (2007)	Brazil (Caucasian)	30	46	14	Population	70	102	38	0.61	(31)
Havemose-Poulsen (2007)	Denmark (Caucasian)	45	63	27	Hospital	25	38	12	0.63	(30)
Ren (2008)	China (Asian)	57	106	8	Population	57	99	15	0.24	(34)
Guzeldemir (2008)	Turkey (Caucasian)	31	38	24	Population	31	51	11	0.23	(33)
Karasneh (2011)	Jordan (Caucasian)	80	101	59	Population	80	106	54	0.15	(35)
Schulz (2011)	Germany (Caucasian)	85	125	45	Population	89	126	52	0.76	(36)
Shibani (2011)	Syria (Caucasian)	32	40	24	Population	35	52	18	0.54	(37)
Fiebig (2008)	Germany/Netherlands (Caucasian)	415	0.84^a^ (0.66–1.06)^a^		Population	874	1.44^b^ (0.93–2.21)^b^		0.52	(32)

a Odds ratio and its 95% confidence interval for T allele vs. G allele;

b odds ratio and its 95% confidence interval for G allele vs. T allele.

HWE, Hardy-Weinberg equilibrium; Mixed, hospital- and population-based.

**Table II:** Results of overall and subgroup analyses of pooled ORs and 95% CIs.

Comparison	Category	Number of studies	OR (95% CI)	P-value of OR	I^2^ (%)	Egger’s P-value
T vs. C	Overall	19	1.00 (0.88–1.14)	0.98	28.86	0.02
	Caucasian	14	0.98 (0.86–1.13)	0.82	29.28	
	Asian	4	1.24 (0.57–2.70)	0.59	53.92	
	African-American	1	1.15 (0.55–2.38)	0.71	0	
	PB	12	0.92 (0.80–1.07)	0.29	34.65	
	HB	5	1.28 (0.91–1.81)	0.16	0	
	Mixed	2	1.34 (0.86–2.09)	0.2	0	
	HWE (Yes)	18	1.03 (0.90–1.18)	0.69	24.39	
	HWE (No)	1	0.65 (0.38–1.09)	0.1	0	
C vs. T	Overall	19	0.99 (0.85–1.14)	0.85	33.66	0.02
	Caucasian	14	1.00 (0.86–1.17)	0.98	35.95	
	Asian	4	0.81 (0.37–1.77)	0.59	53.92	
	African-American	1	0.87 (0.42–1.81)	0.71	0	
	PB	12	1.06 (0.84–1.34)	0.62	41.6	
	HB	5	0.78 (0.55–1.10)	0.16	0	
	Mixed	2	0.75 (0.48–1.17)	0.2	0	
	HWE (Yes)	18	0.95 (0.82–1.11)	0.5	29.34	
	HWE (No)	1	1.55 (0.92–2.62)	0.1	0	

OR, odds ratio; CI, confidence interval; PB, population-based; HB, hospital-based; Mixed, PB and HB; HWE, Hardy-Weinberg equilibrium.
